# Detecting amyloid and tau pathology in Parkinson’s disease, 4R-tauopathies and control subjects with plasma pTau217

**DOI:** 10.3389/fneur.2025.1638852

**Published:** 2025-08-15

**Authors:** Giulia Musso, Eleonora Fiorenzato, Valentina Misenti, Simone Cauzzo, Roberta Biundo, Carmelo A. Fogliano, Giulia Bonato, Wassilios G. Meissner, Marta Campagnolo, Miryam Carecchio, Francesca Vianello, Andrea Guerra, Chiara Cosma, Annachiara Cagnin, Diego Cecchin, Martina Montagnana, Angelo Antonini

**Affiliations:** ^1^Department of Medicine–DIMED, University of Padua, Padua, Italy; ^2^Laboratory Medicine, University-Hospital of Padua, Padua, Italy; ^3^Parkinson and Movement Disorders Unit, Study Center for Neurodegeneration (CESNE), Department of Neuroscience, University of Padova, Padua, Italy; ^4^Department of General Psychology, University of Padova, Padua, Italy; ^5^IRCCS San Camillo, Via Alberoni, Venice, Lido, Italy; ^6^Padova Neuroscience Center (PNC), University of Padova, Padua, Italy; ^7^CHU Bordeaux, Service de Neurologie des Maladies Neurodégénératives, IMNc, Place Amélie Raba-Léon, Bordeaux, France; ^8^Univ. de Bordeaux, CNRS, IMN, UMR, Bordeaux, France; ^9^Complex Operative Unit (UOC) of the Psychology, Neurology Hospital division, Padova University Hospital, Padua, Italy; ^10^Department of Neuroscience, University of Padova, Padua, Italy; ^11^Nuclear Medicine Unit, Department of Medicine (DIMED), University of Padova, Padua, Italy

**Keywords:** Parkinson’s disease, Progressive Supranuclear Palsy, mild cognitive impairment, pTau217, amyloid, tau, dementia, blood-based biomarkers

## Abstract

**Introduction:**

Plasma phospho-tau 217 (pTau217) is a biomarker for Alzheimer’s disease (AD) pathology, reflecting amyloid (Aβ) and tau burden, but its role in Parkinson disease (PD) and 4-repeat(4R)-tauopathies remains incompletely understood. We measured plasma pTau217 across the cognitive spectrum of Lewy body diseases (PD, Dementia with Lewy bodies [DLB]) and in 4R-tauopathies, comparing these groups to cognitively unimpaired (CU) and mild cognitive impairment (MCI) individuals.

**Methods:**

Participants included 18 cognitively normal PD (PD-NC), 32 PD with MCI, and 7 PD with dementia (PDD), alongside 4 DLB patients, grouped as PDD/DLB. The 4R-tauopathy group included 28 Progressive Supranuclear Palsy (PSP) and 4 corticobasal syndrome (CBS) patients, compared to 51 CU and 26 MCI individuals. Ptau217 was measured using the fully automated Lumipulse platform, with values adjusted for creatinine levels. Further, the presence of AD-pathology was defined using a validated cut-off based on Aβ-PET.

**Results:**

PTau217 levels were significantly lower in PD-NC and CU individuals compared to those with greater cognitive impairment (PD-MCI, PDD/DLB, and PSP/CBS), and MCI individuals. AD co-pathology was identified in 28% of PDD/DLB and PSP/CBS patients, 16% of PD-MCI, and none of PD-NC. MCI showed the highest pTau217 positivity (35%), while 8% of CU individuals were positive despite normal cognition. In PD, pTau217 negatively correlated with cognitive performance, as assessed by Montreal Cognitive Assessment (MoCA: *rs* = −0.38, *p* = 0.004) and Mini-Mental State Examination (MMSE: *rs* = −0.37, *p* = 0.006).

**Discussion:**

Plasma pTau217 levels serve as a scalable, non-invasive marker of AD-pathology across Lewy body diseases, PSP/CBS, and MCI/CU populations. AD co-pathology independently contributes to cognitive deficits in PD, but not in PSP/CBS.

## 1 Introduction

The hallmark of Parkinson’s disease (PD) is the presence of alpha-synuclein pathology but *β*-amyloid (Aβ) co-pathology is also often observed (1, 2). Studies indicate that Alzheimer’s disease (AD) co-pathology, a well-documented finding in recent positron emission tomography (PET) and neuropathological studies (3), independently contributes to the development of cognitive deficits and accelerates progression to dementia (1).

In AD, current diagnostic criteria take into account biological markers, either cerebrospinal fluid (CSF), Aβ-PET as well as plasma (4), to detect pathology before cognitive deficits become clinically manifest. However, while the application of blood-based AD biomarkers in PD is still under investigation, it may offer valuable insights for prognosis assessment and early intervention (5, 6).

Phosphorylated-tau (pTau) has demonstrated significant potential as a biomarker for Aβ-pathology, given its strong association with CSF and PET positivity (7–10). Various pTau isoforms, differentiated by their specific phosphorylation sites, have been identified for their capacity to detect Aβ-positive individuals (7, 9, 10). Prominent examples include pTau181, pTau217, pTau231 and pTau235 (7). Among these, pTau217 has emerged as the most reliable and robust marker for Aβ-positivity, offering superior diagnostic accuracy and prognostic utility in the early detection of AD, showing comparable accuracy to CSF biomarkers and an optimal correlation with Aβ (8, 11), and tau PET tracers (8). Plasma pTau217 reflects both Aβ plaque and tau tangle accumulation, serving as an early physiological indicator of Aβ-pathology (12). Notably, plasma pTau217 level may not only predict brain Aβ and tau status but also reflect the topographic extent and magnitude of tau aggregation across the clinical AD spectrum, enabling the discrimination of individuals at intermediate/advanced vs. early AD-stages (8).

The practical advantages of blood-based biomarkers—such as their accessibility, minimally invasive nature, and lower cost compared to CSF or PET—have driven their adoption in both research and clinical settings, for the development of diagnostic algorithms to capture Aβ-pathology at the screening level of mild cognitive impairment (MCI) (13). Plasma pTau217 has shown promise not only in diagnosing AD but also in identifying AD co-pathology in other neurodegenerative disorders, particularly in movement disorders (14). For instance, some preliminary studies has demonstrated utility in Lewy body dementias (PDD and DLB) (5, 15) as well as in 4R-tauopathies, including Progressive Supranuclear Palsy (PSP) and corticobasal syndrome (CBS) (12).

Herein, for the first time to the best of our knowledge, we measured plasma pTau217 in Lewy body diseases with various degree of cognitive dysfunctions—ranging from cognitively normal PD (PD-NC), PD-MCI, PDD and DLB—and in 4R-tauopathies (PSP and CBS) (16), comparing these results relative to cognitively unimpaired (CU) and MCI individuals. Further, we adopted a pTau217 cut-off, previously validated against Aβ-PET imaging, as a proxy of AD pathology to determine its presence (17).

## 2 Materials and methods

### 2.1 Study participants

From the PADUA-CESNE cohort, we included a total of 170 participants recruited at the Parkinson Disease and Movement Disorders Unit of Padua University Hospital (Padua, Italy).

The cohort comprises 93 patients: 57 PD patients with various degree of cognitive dysfunction and four with a probable diagnosis of DLB, according to recent criteria (18, 19). PD patients underwent a II-level cognitive assessment, and were classified as cognitively normal (PD-NC, *n* = 18), with MCI (PD-MCI, *n* = 32) and dementia (PDD, *n* = 7). PDD were combined with DLB into a single group, reflecting the continuum of Lewy body dementias (PDD/DLB, *n* = 11) (20). Although clinical, neuropathological, and genetic differences exist between PDD and DLB, substantial overlap is well recognized (21). Furthermore, recent evidence indicates no differences in plasma pTau217 concentrations between these subgroups (5, 15).

Regarding the 4R-tauopathy group, this group included 28 patients with probable PSP (the phenotypes are detailed in Supplementary material), and four withprobable CBS—referred as the “PSP/CBS” group (16, 22, 23). This grouping was also based on previous evidence showing no statistically significant differences in pTau217 concentrations, despite a greater variability in CBS vs. PSP patients (16).

All the patients included in the study were diagnosed by movement disorder specialists, underwent MRI, and were followed up for at least 2 year for the diagnostic work-up and on-going clinical/cognitive assessment. The Hoehn and Yahr (H&Y) scale was adopted to assess disease severity, and the levodopa (LEDD) and the dopamine agonist equivalent daily dosages (DAED) were calculated for each treated patient.

Furthermore, as part of Italian National Recovery Fund “AGE-it” project on normal aging,^1^ we recruited 77 control subjects without major medical comorbidities. Participants with a neuropsychological assessment performance within 1.5 standard deviation (SD) of the published norms for their age group, were classified as CU; whereas a classification of MCI was determined based on internationally agreed clinical criteria. The cohort included 51 CU participants, and 26 with MCI (24).

Exclusion criteria for the current study included the presence of MRI abnormalities (such as cerebral vascular lesions, diffuse white matter hyperintensities, meningiomas, brain cysts or tumors or other space-occupying lesions), a history of head injuries, and any significant psychiatric, neurological, or systemic comorbidities.

All subjects underwent a cognitive evaluation, including the assessment of global cognition by means of Montreal Cognitive Assessment (MoCA) and Mini-Mental Scale Examination (MMSE) scales (25), with scores adjusted for age and education according to published norms. PD patients underwent a II-level comprehensive cognitive assessment, specifically designed to target PD-cognitive deficits, allowing to classify patients across the entire PD-cognitive spectrum, while CU and MCI individuals were evaluated with a I-level assessment and a questionnaire of subjective cognitive complaints (26). Of note, the PD cognitive diagnosis was additionally verified using a Level-I assessment to ensure consistency in the diagnostic criteria applied across groups (Level-I vs. Level-II), with no differences observed in cognitive classification (27). The adopted cognitive battery is detailed in Supplementary material, and in previous works (25, 28). The study protocol was approved by the local ethics committee at Padua University Hospital and conducted according to the Declaration of Helsinki. All study participants gave written informed consent.

### 2.2 Plasma pTau217

pTau217 was tested in K-2 ethylenediaminetetraacetic acid plasma samples with a research-use-only Lumipulse G1200 assay (Fujirebio, Japan; lot 4,129). Samples were aliquoted in polypropylene tubes and kept frozen at −80°C and handled as previously reported before analyses (29); hemolyzed samples were excluded due to potential interference effect (30). Manufacturer’s quality controls lot number D6C5055 (level 1 target mean 0.488 ± 0.098, level 2 target mean 3.997 ± 0.799) were tested in each analytical run, with a coefficient of variation <10% for both levels. To detect the presence of AD-pathology using plasma pTau217 as a proxy, we applied a previously validated cutoff (established against Aβ-PET using the same assay), accounting for a maximum analytical variability of 10% (17). Therefore, patients were considered positive for AD-pathology, when having a pTau217 above 0.22 ng/L.

### 2.3 Statistical analyses

The Shapiro–Wilk test was used to assess the normality of the data distribution. Categorical variables and frequencies were analyzed using the chi-square test. For non-normally distributed data, Kruskal-Wallis test was applied, followed by pairwise comparisons using the Dwass-Steel-Critchlow-Fligner method. For normally distributed data, analysis of variance (ANOVA) or covariance (ANCOVA) models were adopted. Between-group differences in plasma pTau217 levels were assessed through an ANCOVA model. Log-transformed pTau217 values were used to normalize the distribution, and creatinine concentrations (Roche Diagnostics, Switzerland) were included as a covariate, given that renal dysfunction—reflected by elevated creatinine levels—can influence pTau217 concentrations (31, 32). Although no statistically significant age differences were observed across diagnostic subgroups, we performed an additional ANCOVA including age—alongside creatinine levels—as a covariate to account for its potential confounding effect. This analysis was further supported by prior studies reporting mixed findings on the association between age and pTau217 levels, with some identifying a relationship (32), and others not (15, 33). To further explore this association, we also performed Spearman correlation analyses between pTau217 levels and age. In addition, partial Spearman correlations were conducted between plasma pTau217 levels and cognitive/clinical features (MoCA, MMSE, disease duration, and H&Y), adjusting for creatinine levels. Lastly, the percentage of pTau217-positive cases and their average pTau217 levels were calculated for each group.

Of note, after evaluating potential significant differences in pTau217 concentrations between groups, given the limited number of CBS patients (*n* = 4), we combined them together with PSP resulting in the primary tauopathies group referred as “PSP/CBS” (16). Likewise, PDD and DLB (*n* = 4) were combined into a single group, reflecting the continuum of Lewy body dementias (15, 20). False Discovery Rate (FDR) correction was applied for multiple comparison corrections. All the statistical analyses were performed using R (version 4.2.3) and IBM SPSS Statistics (version 24); the statistical significance threshold was set at *p ≤* 0.05.

## 3 Results

### 3.1 Sample demographic and clinical features

Demographic, cognitive, clinical features and plasma pTau217 concentrations are summarized in Table 1. No significant differences were noted in age or sex between groups. Although the overall ANOVA model for age was significant, post-hoc tests showed no significant difference between CU and MCI [t(164) = −2.70; *p_FDR_* = 0.114], as well as between the other subgroups.

**Table 1:** Demographic, clinical, and cognitive characteristics of clinical groups.

		CU (*n* = 51)		MCI (*n* = 26)		PD-NC (*n* = 18)		PD-MCI (*n* = 32)		PDD/ DLB (*n* = 11)		PSP/ CBS (*n* = 32)		Total sample (*n* = 170)		
		Mean (SD)	Min-max	Mean (SD)	Min-max	Mean (SD)	Min-max	Mean (SD)	Min-max	Mean (SD)	Min-max	Mean (SD)	Min-max	F (df1, df2)/χ^2^ (df)	*p*-value	Post-hoc
Age		70.8 (3.71)	64–80	74.31 (5.68)	65–84	71.11 (5.83)	63–87	73.72 (5.88)	63–82	71.73 (6.34)	58–80	73.06 (6.22)	56–85	2.27 (5, 164)	**0.05**	
Sex, f/m		26/25		13/13		8/10		14/18		2/9		13/19		4.47 (5)	0.485	
Disease duration, yrs						8.44 (5.88)	1–19	9.16 (6)	1–23	5.91 (4.5)	1–14	3.88 (2)	1–9	7.41 (3, 88)	**<0.001**	j, l
pTau217, ng/L	§	0.12 (0.15)	0.03–1.05	0.22 (0.24)	0.04–0.91	0.08 (0.03)	0.04–0.18	0.15 (0.12)	0.03–0.57	0.16 (0.1)	0.05–0.39	0.17 (0.11)	0.03–0.55	21.38 (6)	**<0.001**	
Creatinine, mmol/L		76.98 (14.42)	53–115	75.08 (15.04)	51–112	67.17 (16.67)	37–106	66.13 (20.51)	31–114	71.09 (10.72)	53–85	69.19 (15.44)	35–101	2.55 (5,164)	**0.030**	b
pTau217, pos/neg (% positivity)		4/47 (7.84%)		9/17 (34.62%)		0/18 (0%)		5/27 (15.63%)		3/8 (27.27%)		9/23 (28.13%)		15.59 (5)	**0.008**	a, d, e, i, j
pTau217, ng/L (of positive cases)	§	0.53 (0.36)	0.22–1.05	0.47 (0.26)	0.22–0.91	-	-	0.38 (0.13)	0.24–0.57	0.29 (0.08)	0.24–0.39	0.30 (0.11)	0.22–0.55	5.23 (4)	0.264	
MoCA	§	25.9 (2.71)	18–30	22.08 (3.47)	14–28	26 (2.3)	23–30	22.06 (3.16)	15–27	16.67 (3.84)	13–22	18.66 (4.25)	9–26	79.74 (5)	**<0.001**	a, b, c, d, f, g, h, i, j, k, l
MMSE	§	28.96 (1.17)	25–30	28.19 (1.5)	24–30	29.17 (0.99)	27–30	27.34 (1.64)	22–30	24.33 (3.08)	20–29	26.03 (2.82)	18–30	57.01 (5)	**<0.001**	b, c, d, f, g, h, i, j
H&Y	§					1.92 (0.81)	1–4	2.39 (0.83)	1–5	2.5 (1.14)	1–5	3.52 (1.12)	1–5	28.57 (3)	**<0.001**	j, l, m
LEDD	§					594.91 (472.2)	0–1,720	448.54 (245.05)	56–1,010	543.57 (486.7)	0–1,150			0.42 (2)	0.811	
DAED	§					92.06 (105.45)	0–320	47.1 (74.23)	0–320	15 (39.69)	0–105			4.67 (2)	0.097	

Significant *p*-values are shown in bold. §, not normally distributed variables; SD; standard deviation; MoCA, Montreal Cognitive Assessment; MMSE, Mini-mental State Examination; H&Y, Hoehn and Yahr Scale; LEDD, levodopa equivalent daily dose; DAED, dopamine agonist equivalent daily dose. Significant differences after multiple comparison corrections: (a) CU vs. MCI; (b) CU vs. PD-MCI; (c) CU vs. PDD/DLB; (d) CU vs. PSP/CBS; (e) MCI vs. PD-NC; (f) MCI vs. PDD/DLB; (g) MCI vs. PSP/CBS; (h) PD-NC vs. PD-MCI; (i) PD-NC vs. PDD/DLB; (j) PD-NC vs. PSP/CBS; (k) PD-MCI vs. PDD/DLB; (l) PD-MCI vs. PSP/CBS; (m) PDD/DLB vs. PSP/CBS.

**Figure 1:**
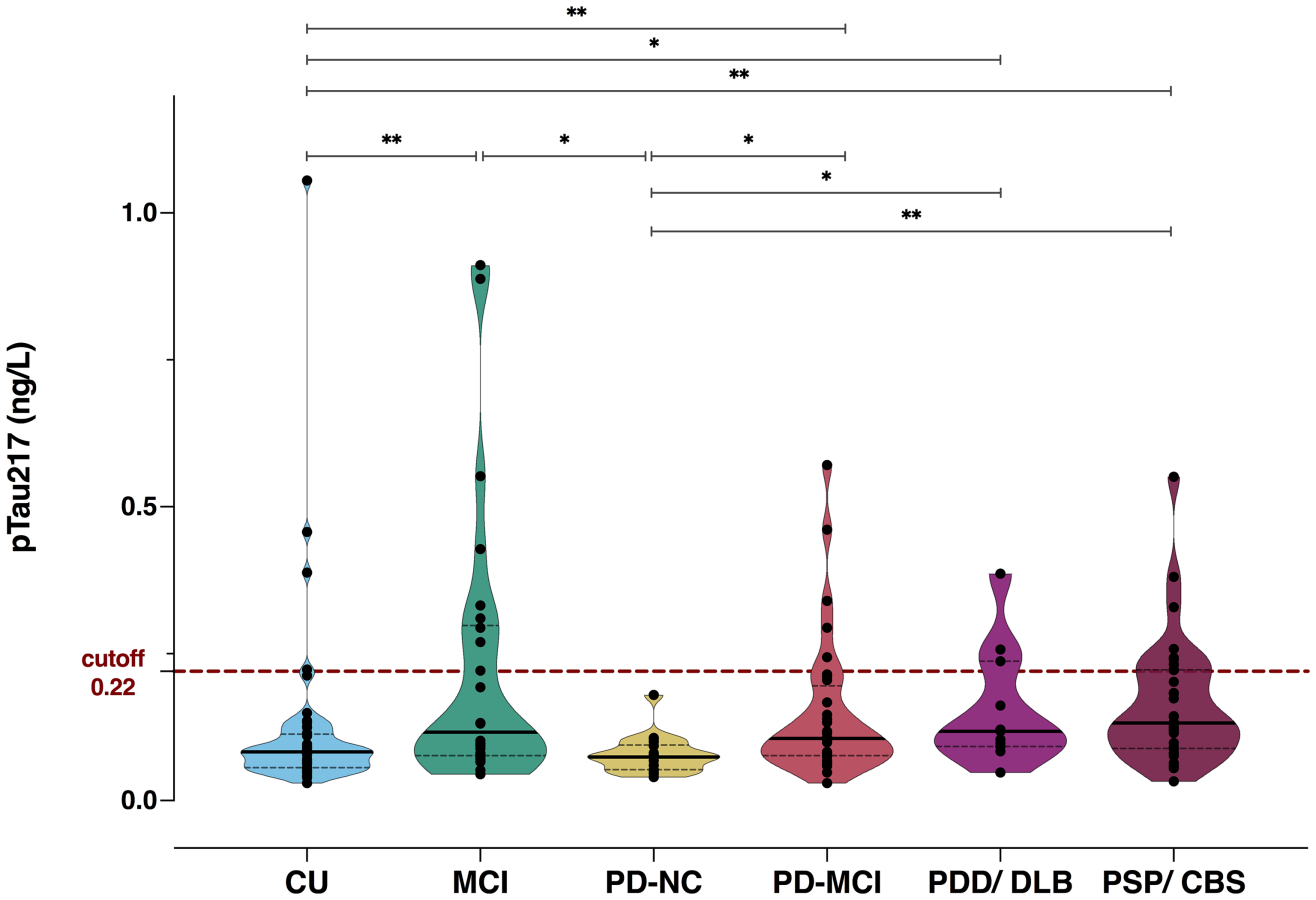
Plasma pTau217 levels across clinical groups. CU, cognitively unimpaired; MCI, mild cognitive impairment; PD, Parkinson’s disease; NC, normal cognition; PDD, PD with dementia; DLB, Dementia with Lewy bodies; PSP, Progressive Supranuclear Palsy; CBS, corticobasal syndrome. * < 0.05; ** < 0.01.

Plasma pTau217 concentrations significantly differed across groups (Figure 1). Namely, CU individuals showed lower pTau217 than MCI [t(163) = −3.72, *p_FDR_* = 0.002], PD-MCI [t(163) = −3.08, *p_FDR_* = 0.007], PDD/DLB [t(163) = −2.33, *p_FDR_* = 0.046] and PSP/CBS patients [t(163) = −3.96, *p_FDR_* = 0.002] with the exclusion of PD-NC; while MCI had higher levels of pTau217 than PD-NC [t(163) = 3.20, *p_FDR_* = 0.006]. PD-NC showed lower pTau217 than PD-MCI [t(163) = −2.73, *p_FDR_* = 0.017], PDD/DLB [t(163) = −2.27, *p_FDR_* = 0.046] and PSP/CBS [t(163) = −3.39, *p_FDR_* = 0.004]. Of note, creatinine levels were significantly higher in MCI individuals compared to CU [t(164) = −3.00, *p_FDR_* = 0.048], with no differences observed in other group comparisons. The additional ANCOVA model, which includes age alongside creatinine as covariates, further confirmed our findings, as the results remained consistent when controlling for age (see Supplementary material).

**Figure 2:**
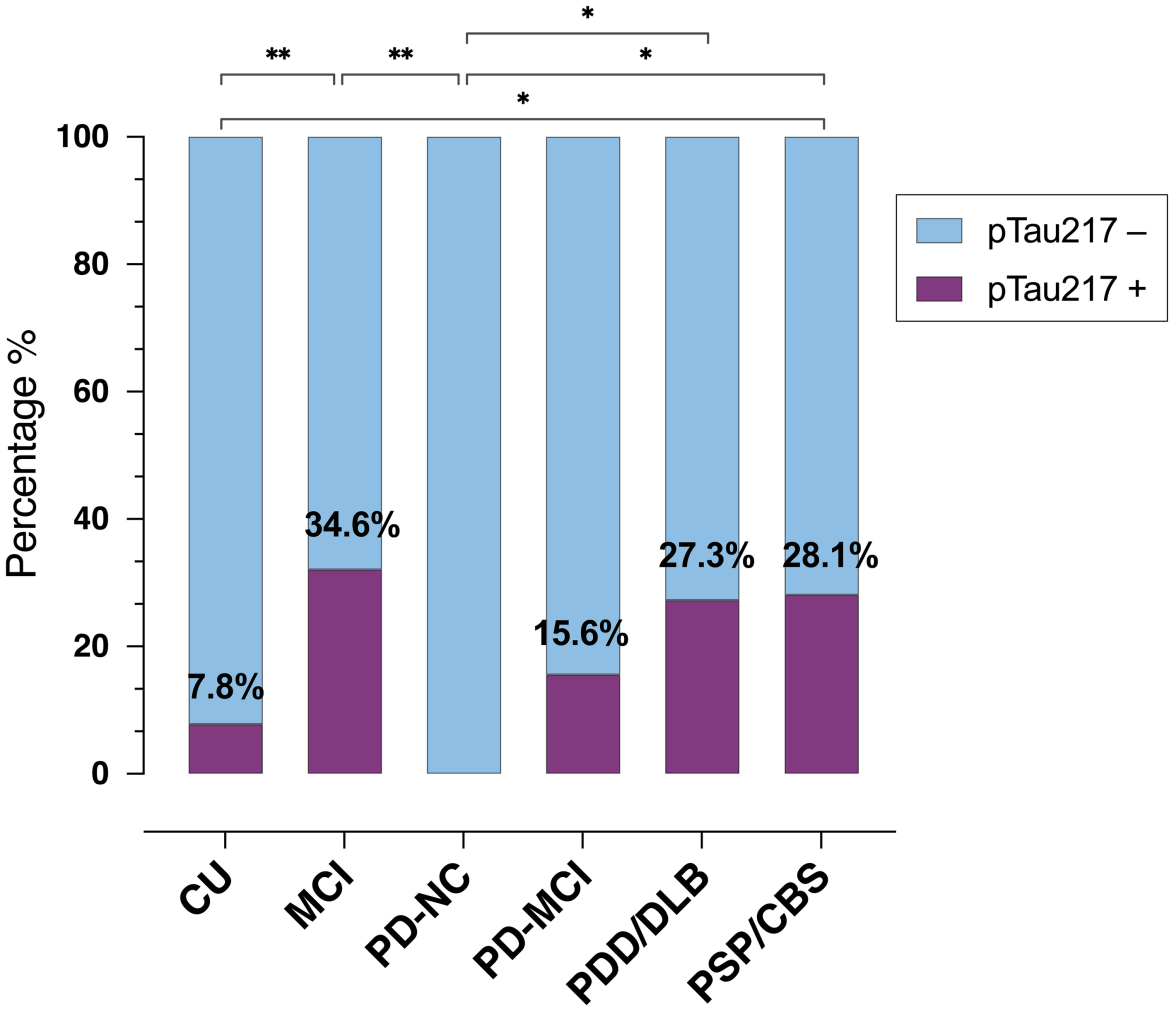
Prevalence of plasma pTau217-positive cases across groups. CU, cognitively unimpaired; MCI, mild cognitive impairment; PD, Parkinson’s disease; NC, normal cognition; PDD, PD with dementia; DLB, Dementia with Lewy bodies; PSP, Progressive Supranuclear Palsy; CBS, corticobasal syndrome. * < 0.05; ** < 0.01.

As shown in Figure 2, the percentage of pTau217-positive cases (>0.22 ng/L) varied significantly across groups (*p* = 0.008), with the highest percentage in MCI (34.6%, *n* = 9), CBS/PSP (28.1%, *n* = 9) and PDD/DLB (27.3%, *n* = 3) groups, followed by PD-MCI (15.6%, *n* = 5) and CU (7.8%, *n* = 4). No pTau217-positive cases were observed in PD-NC. The proportion of pTau217-positive cases significantly differed between PD-NC and the following groups: MCI [χ^2^(1) = 7.66; *p =* 0.006], PDD/DLB [χ^2^(1) = 5.29; *p* = 0.021], and PSP/CBS [χ^2^(1) = 6.05; *p =* 0.014]. CU individuals had fewer pTau217-positive cases than MCI and PSP/CBS [χ^2^(1) = 8.69; *p =* 0.003, and χ^2^(1) = 6.05; *p =* 0.014, respectively], while the CU vs. PDD/DLB comparison showed only a trend [χ^2^(1) = 3.36; *p =* 0.07], likely due to the small PDD/DLB sample size.

Regarding disease duration, significant differences were observed between patient groups. Both PD-NC and PD-MCI had longer disease durations than PSP-CBS [t(88) = 3.26, *p =* 0.009 and t(88) = 3.26, *p* < 0.001, respectively]. Motor severity, assessed by Hoehn and Yahr (H&Y), was more pronounced in PSP/CBS patients than all PD subgroups (PD-NC: W = 6.13, *p* < 0.001; PD-MCI: W = 6.00, *p* < 0.001 and PDD/DLB: W = 3.73, *p =* 0.041). No significant differences were observed in daily dosages of levodopa and dopamine agonists across PD subgroups.

Global cognitive functioning, assessed by the Montreal Cognitive Assessment (MoCA), revealed greater cognitive impairments in all disease groups compared to the CU group (all *p* < 0.001), with the exception of PD-NC. PDD/DLB and PSP/CBS exhibited more severe cognitive impairments than PD-MCI (both *p =* 0.015), PD-NC (both *p* < 0.001), and MCI (*p =* 0.015 and *p =* 0.028, respectively). Lastly, as expected, PD-MCI scored lower than PD-NC patients (*p* < 0.001). Similar patterns were observed in Mini-Mental State Examination (MMSE) scores, with PD-MCI, PDD/DLB, and PSP/CBS performing worse global than CU individuals (all *p* < 0.001). MCI exhibited less cognitive impairment than PDD/DLB and PSP/CBS (*p =* 0.025 and *p =* 0.015, respectively), while PD-NC performed better than PD-MCI (*p* < 0.001), PDD/DLB (*p =* 0.005), and PSP/CBS (*p* < 0.001).

### 3.2 Correlations between pTau217, demographic, cognitive and clinical features

Regarding the association between pTau217 and age, a significant positive correlation was observed only within the PD subgroup (*rs* = 0.37, *p =* 0.004), while no significant correlations were found in the CU (*rs* = 0.023, *p* = 0.873), MCI (*rs* = 0.083, *p* = 0.688) or PSP (*rs* = 0.265, *p* = 0.173) subgroups.

**Figure 3:**
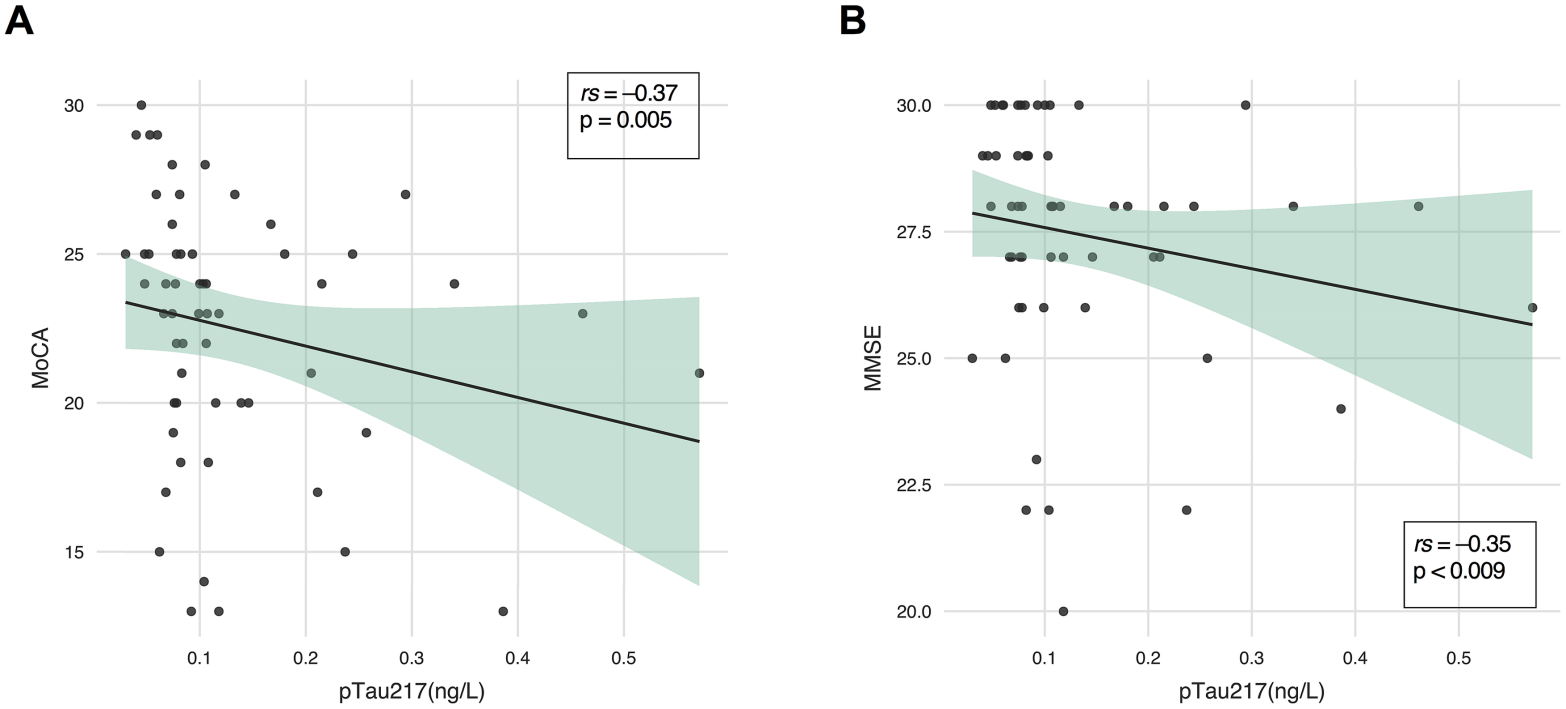
Correlation between plasma pTau217 levels and cognitive performance within the PD group. Plasma pTau217 correlation with **(A)** Montreal Cognitive Assessment (MoCA), and **(B)** Mini-Mental State Examination (MMSE) in Parkinson’s disease (PD) (*n* = 57).

In the overall PD group (n = 57), pTau217 levels negatively correlated with global cognitive functioning, assessed by both MoCA (*rs* = −0.37, *p =* 0.005) and MMSE (*rs* = −0.35, *p =* 0.009) (Figures 3A,B). In contrast, no statistically significant correlations were found between pTau217, and either disease duration or motor severity, as measured by the H&Y scale, in both PD and PSP groups (all *p* > 0.05).

Furthermore, in the CU, MCI, and PSP/CBS groups, no statistically significant correlations were found between pTau217 and global cognitive functioning (MMSE and MoCA).

## 4 Discussion

We measured plasma pTau217 levels—a biomarker specific to AD co-pathology—in PD patients with varying degrees of cognitive dysfunction (including DLB), as well as in PSP/CBS, and compared them to MCI and cognitively normal individuals.

Our main finding is that plasma pTau217 concentrations (adjusted for creatinine) were significantly more elevated in PD subgroups with greater cognitive impairment (PD-MCI, PDD/DLB, and PSP/CBS), and in individuals with MCI, compared to PD-NC and CU. Furthermore, using a previously validated pTau217 cut-off (against Aβ-PET data) as a proxy of AD pathology (17), we documented AD co-pathology in approximately 28% of PDD/DLB and PSP/CBS patients and 16% of PD-MCI. No positive cases were observed among PD-NC individuals. These findings, consistent with ours and other previous reports, seem to suggest a relationship between increasing AD co-pathology and worse PD-cognitive status (2, 34–36).

As expected, the highest prevalence of AD-pathology was observed in the MCI group, with 35% of individuals testing pTau217-positive, while 8% of the CU group were also positive despite normal cognitive performance, suggesting the presence of prodromal AD cases. This is not surprising, but is aligned with previous evidence, which interprets elevated pTau as an early physiological reaction to brain Aβ plaque deposition, preceding the widespread aggregation of neurofibrillary tangles (8). In this regard, plasma pTau217 has demonstrated prognostic utility in predicting cognitive decline over a 2-year period in individuals with MCI, and also in cognitively unimpaired individuals, highlighting its potential value as a screening measure for early AD detection, before the use of more invasive and costly CSF/PET techniques (37–39).

Hence, our results support plasma pTau217 as a promising marker for identifying individuals at risk of cognitive decline—not only in elderly populations, but also in degenerative parkinsonisms.

A further observation emerges when analyzing only pTau217-positive individuals: the average concentrations in parkinsonian disorders with cognitive impairment (PD-MCI, PDD/DLB, PSP/CBS) ranged from 0.29 to 0.38, notably lower than those reported in an AD cohort (about 0.66), as documented in our previous study using the same Lumipulse G assay (17). These findings converge with prior PET (2), and neuropathological studies (40), revealing a lower Aβ/tau burden in PD compared to AD irrespective of age of the patient and disease duration. Although the exact underlying biological mechanisms remain unclear, this lower burden in synucleinopathies may result from modulatory and interacting processes between Lewy bodies and Aβ, potentially affecting the pathological spread of Aβ as compared to “pure AD” (40).

We also found that plasma pTau217 levels were negatively correlated with global cognitive functioning in PD, as assessed by MoCA and MMSE scales, indicating that AD co-pathology, albeit less pronounced, might aggravate cognitive dysfunctions in synucleinopathies, contributing to a worse cognitive profile in PD (3, 35, 36). Similar associations have been reported by a recent first report demonstrating a relationship between plasma p-tau217 and MoCA performance in a cohort of PDD/DLB patients and PD at low-dementia risk (15). This convergence strengthens the case for the superiority of plasma pTau217 as a more specific and sensitive biomarker of cognitive deterioration compared to other plasma markers (e.g., pTau181, Aβ42/40, and neurofilament light chain), suggesting its potential utility in both clinical and research settings.

Conversely, in the PSP/CBS group, despite having more severe global cognitive dysfunctions than PD-MCI and PD-NC (41), pTau217 levels did not correlate with cognitive performance. This may underscore the distinct pathological mechanisms in PSP, where cognitive deficits are primarily linked to cortical 4R-tau pathology rather than AD co-pathology (42). Although Aβ accumulation seems relatively common in PSP, it appears to be rather associated with advanced age and minimally contributes to PSP-related motor and cognitive impairment (43).

A notable but controversial finding was the lack of a significant correlation between plasma pTau217 levels and global cognition in the MCI group, despite its higher prevalence of pTau217-positivity. Previous longitudinal studies have demonstrated that steeper increases in pTau217 correlate with accelerated MMSE decline, pointing to the need for prospective investigation in larger cohorts (44).

Lastly, we observed a significant association between pTau217 and age only within the PD subgroup, which differs from findings reported in a previous study in a PDD/DLB cohort (5). However, it is important to note that also evidence in CU/MCI populations remains inconsistent (32, 33), with recent research emphasizing the need for age-specific thresholds to interpret pTau217 levels, particularly in CU individuals under 65 years (45). Regarding disease progression, in contrast to the AD framework, we found no direct association between pTau217 and clinical progression in either the PD or PSP groups, although this is not unexpected, given that AD pathology is not the primary driver in these conditions (39).

Our study has some limitations. First, given that Aβ status was not confirmed by PET imaging or CSF analysis, pTau217 in this study can only be regarded as a proxy of AD pathology, rather than as evidence of confirmed AD co-pathology in our sample. However, a robust plasma Lumipulse pTau217 cutoff (>0.22 ng/L) was adopted [ROC AUC was 0.99 (CI: 0.98–1)], which we previously validated in a cohort of cognitively impaired subjects while adopting the same methodology (17). Furthermore, the use of a similar threshold is also supported by a recent head-to-head comparison of different pTau assays (46), and by another study using the same fully automated Lumipulse platform (47, 48).

Additional limitations of this study include the small sample size, cross-sectional design, and lack of neuropathological confirmation. Namely, to address sample sizes constraints, we combined some diagnostic subgroups based on previous evidence reporting no significant differences in pTau217 concentrations—namely, PDD and DLB were analyzed together as Lewy Body disorders (5, 15), while CBS (*n* = 4) was grouped with PSP (16). We recognize that combining distinct disease entities may mask disease-specific patterns; therefore, our findings—particularly those involving the CBS subgroup—should be interpreted as exploratory. In addition, the small sample sizes in certain disease subgroups likely limited statistical power and may reduce the generalizability of our findings. The lack of longitudinal data also constrains our ability to draw firm conclusions about the causal impact of AD co-pathology on cognitive decline. Lastly, we acknowledge that chronic kidney disease can affect pTau217 concentrations, and given that CU vs. MCI groups differed for creatinine levels, we adjusted plasma pTau217 by including creatinine levels as covariate in the statistical models (31, 32).

In conclusion, plasma pTau217 levels, as a proxy for both Aβ and tau pathology, represent a scalable, cost-effective, and non-invasive marker of AD pathology across the PD-cognitive spectrum, PSP/CBS, and MCI/CU populations, to timely identify individuals at risk of cognitive deterioration.

AD co-pathology, despite of reduced magnitude compared to AD, seem to independently contribute to cognitive decline in PD, but not in PSP patients. Longitudinal studies with larger cohorts are warranted to further confirm the role of plasma pTau217 measures in Lewy body diseases and in 4R-tauopathies.

## Data Availability

The raw data supporting the conclusions of this article will be made available by the authors, without undue reservation.

## Footnotes

^1^ https://ageit.eu/wp/en/
