# Supplementary material for: Detecting amyloid and tau pathology in Parkinson’s disease, 4R-tauopathies and control subjects with plasma pTau217
Source: Front Neurol. 2025 Aug 15;16:1638852. doi: 10.3389/fneur.2025.1638852 (PMC12395377; doi:10.3389/fneur.2025.1638852)
Supplement: Supplementary file 1 [file Presentation_1.pdf]

Supplementary materials

1. SUPPORTING METHODS

1.1 Participants: Clinical and cognitive assessment

From the PADUA-CESNE cohort, we included a total of 170 participants recruited at the Parkinson Disease and Movement Disorders Unit of Padua University Hospital (Padua, Italy). Among the recruited patients, 28 had a diagnosis of probable PSP, whose phenotypes were defined based on the MDS diagnostic criteria:[1] 12 PSP with Richardson Syndrome (PSP-RS), 7 PSP- Parkinsonism (PSP-P), 4 PSP-CBS, 3 PSP with the Frontal Variant (PSP-F), 1 PSP with the Primary Gait Freezing (PSP-PGF) and 1 PSP with Speech-Language Variant (PSP-SL).

1.2 Neuropsychological battery

The II-level neuropsychological assessment evaluates six cognitive domains—memory, executive functions, attention/working memory, visuospatial abilities, language, and social cognition—based on the criteria outlined in the Diagnostic and Statistical Manual of Mental Disorders (DSM-5) and the MDS guidelines.[2–4] Memory was assessed using the Prose Memory Test, and the Rey Auditory Verbal Learning Test (RAVLT) and the delayed copy of Rey–Osterrieth Complex Figure (ROCF).[5] Executive functions were evaluated through the Stroop Test,[6] Clock Drawing Test,[7] as well as the phonemic verbal fluency task.[8] Attention/working memory were assessed using the Trail Making Test (B-A),[9] and the Symbol Digit Modality Test (SDMT, verbal version)[10] and the alternate verbal fluency task.[8] Visuospatial abilities were measured by the Judgment of Line Orientation (Benton JLO),[11] and the immediate copy of ROCF.[12] Language was assessed through category verbal fluency,[8] and the Boston Naming Test.[13]

Social cognition was evaluated using the Story-Based Empathy Task,[14] and the FACE test.[15]

In contrast, the I-level neuropsychological examination included at least one test per cognitive domain. The selected tests for this level were the verbal fluency tasks (phonemic, semantic, and alternate), the FACE test, the RAVLT, the SDMT, the Benton JLO, and the Story-Based Empathy Task. Additionally, the Multidimensional Assessment of Subjective Cognitive Decline (MASCoD) scale was incorporated to assess self-reported cognitive decline.[16]

Parkinson's disease (PD) patients underwent a II-level cognitive assessment, and z-scores were calculated for each test and individual, using standardized Italian norms that account for age and education. PD patients were classified as having mild cognitive impairment (PD-MCI) if their z-scores for a given test were at least 1.5 standard deviations (SD) below the appropriate normative values on two tests—either within a single cognitive domain or on at least one test across two or more cognitive domains.[2] The presence of Parkinson's disease dementia (PDD) was assessed according to the MDS Task Force recommendations,[3] which included evaluations of cognitive function, daily living activities, and neuropsychiatric status. Patients without cognitive impairment were classified as having Parkinson's disease with normal cognition (PD-NC). Similarly, healthy older participants underwent an I-level cognitive assessment, and the presence of MCI was determined according to DSM-5 criteria and established MCI guidelines.[4, 17] These included: i) the presence of cognitive complaints (by the participant or informant); ii) objective evidence of impairment in at least one cognitive domain (defined as 1.5 SD below the mean of published norms), iii) no clinically significant impairment in activities of daily living, and iv) absence of dementia. In addition, to objectively measure the subjective cognitive complaints, a score of 8 or higher on the MASCoD was considered.

2. SUPPORTING RESULTS

2.1 Plasma pTau217 across groups

Plasma pTau217 concentrations significantly differed across groups, also when including age as covariate alongside creatinine levels. Namely, CU individuals showed lower pTau217 than MCI (t(162) = –3.17, $p_{FDR} = 0.009$), PD-MCI (t(162) = –2.56, $p_{FDR} = 0.034$), PDD/DLB (t(162) = –2.24, $p_{FDR} = 0.050$) and PSP/CBS patients (t(162) = –3.58, $p_{FDR} = 0.007$) with the exclusion of PD-NC; while MCI had higher levels of pTau217 than PD-NC (t(162) = 2.89, $p_{FDR} = 0.016$). PD-NC showed lower pTau217 than PD-MCI (t(162) = –2.44, $p_{FDR} = 0.040$), PDD/DLB (t(162) = –2.26, $p_{FDR} = 0.050$) and PSP/CBS (t(162) = –3.20, $p_{FDR} = 0.009$).

3. REFERENCES

1. Höglinger GU, Respondek G, Stamelou M, et al (2017) Clinical diagnosis of progressive supranuclear palsy: The movement disorder society criteria: MDS Clinical Diagnostic Criteria for PSP. Mov Disord 32:853–864. https://doi.org/10.1002/mds.26987

2. Litvan I, Goldman JG, Tröster AI, et al (2012) Diagnostic criteria for mild cognitive impairment in Parkinson's disease: Movement Disorder Society Task Force guidelines. Movement Disorders 27:349–356. https://doi.org/10.1002/mds.24893

3. Emre M, Aarsland D, Brown R, et al (2007) Clinical diagnostic criteria for dementia associated with Parkinson's disease. Mov Disord 22:1689–707; quiz 1837. https://doi.org/10.1002/mds.21507

4. APA (2012) Diagnostic and Statistical Manual DSM 5. American Psychiatric Pub, Washington

5. Gasparini M, Scandola M, Amato S, et al (2024) Normative data beyond the total scores: a process score analysis of the Rey's 15 word test in healthy aging and Alzheimer's Disease. Neurol Sci 45:2605–2613. https://doi.org/10.1007/s10072-024-07330-0

6. Caffarra P, Vezzadini G, Dieci F, et al (2002) Una versione abbreviata del test di Stroop: dati normativi nella popolazione italiana. Nuova Rivista di Neurologia 12:111–115

7. Siciliano M, Santangelo G, D'Iorio A, et al (2016) Rouleau version of the Clock Drawing Test: age- and education-adjusted normative data from a wide Italian sample. Clin Neuropsychol 30:1501–1516. https://doi.org/10.1080/13854046.2016.1241893

8. Costa A, Bagoj E, Monaco M, et al (2014) Standardization and normative data obtained in the Italian population for a new verbal fluency instrument, the phonemic/semantic alternate fluency test. Neurol Sci 35:365–372. https://doi.org/10.1007/s10072-013-1520-8

9. Giovagnoli AR, Del Pesce M, Mascheroni S, et al (1996) Trail making test: normative values from 287 normal adult controls. Ital J Neurol Sci 17:305–9

10. Nocentini U, Giordano A, Di Vincenzo S, et al (2006) The Symbol Digit Modalities Test - Oral version: Italian normative data. Funct Neurol 21:93–96

11. Gullett JM, Price CC, Nguyen P, et al (2013) Reliability of three Benton Judgment of Line Orientation short forms in idiopathic Parkinson's disease. The Clinical Neuropsychologist 27:1167–1178

12. Caffarra P, Vezzadini G, Dieci F, et al (2002) Rey-Osterrieth complex figure: normative values in an Italian population sample. Neurological Sciences 22:443–447

13. Williams BW, Mack W, Henderson VW (1989) Boston Naming Test in Alzheimer's disease. Neuropsychologia 27:1073–1079. https://doi.org/10.1016/0028-3932(89)90186-3

14. Dodich A, Cerami C, Canessa N, et al (2015) A novel task assessing intention and emotion attribution: Italian standardization and normative data of the Story-based Empathy Task. Neurol Sci 36:1907–1912. https://doi.org/10.1007/s10072-015-2281-3

15. Terruzzi S, Funghi G, Meli C, et al (2023) The FACE test: a new neuropsychological task to assess the recognition of complex mental states from faces. Neurol Sci. https://doi.org/10.1007/s10072-023-06697-w

16. Maffoni M, Pierobon A, Fundarò C (2022) MASCoD-Multidimensional Assessment of Subjective Cognitive Decline. Front Psychol 13:921062. https://doi.org/10.3389/fpsyg.2022.921062

17. Dunne RA, Aarsland D, O'Brien JT, et al (2021) Mild Cognitive Impairment: the Manchester consensus. Age and Ageing 50:72–80. https://doi.org/10.1093/ageing/afaa228
